# Supplementary material for: A Bayesian Network Meta-Analysis and Systematic Review of Guidance Techniques in Botulinum Toxin Injections and Their Hierarchy in the Treatment of Limb Spasticity
Source: Toxins (Basel). 2023 Mar 31;15(4):256. doi: 10.3390/toxins15040256 (PMC10145352; doi:10.3390/toxins15040256)
Supplement: Supplementary file 1 [file toxins-15-00256-s001.zip › toxins-2277734-supplementary.pdf]

Supplementary Materials: A Bayesian Network Meta-Analysis and Systematic Review of Guidance Techniques in Botulinum Toxin Injections and Their Hierarchy in the Treatment of Limb Spasticity

Evridiki Asimakidou and Christos Sidiropoulos

Table S1. Muscles injected with botulinum toxin and the corresponding dose/dose range in units (U) of Onabotulinum A. in each study. **Abbreviations:** PMa: pectoralis major, LSC: levator scapulae, TM: teres major,BB: biceps brachii, TBR: triceps brachii, BR: brachialis, BRD: brachioradialis, PT: pronator teres, FCR: flexor carpi radialis, FCU: flexor carpi ulnaris, FDS: flexordigitorum superficialis, FDP: flexor digitorum profundus, FPL: flexor pollicis longus, OP: opponens pollicis, LUM: lumbricales muscles, ILPS: iliopsoas, ADD:adductors, PCT: pectineus, HMST: hamstrings, GASTR: gastrocnemius, SOL: soleus, TP: tibialis posterior.

Authors	PMa	LSC	TM	BB	TBR	BR	BRD	PT	FCR	FCU	FDS	FDP	FPL	OP	LUM	ILPS	ADD	PCT	HMST	GASTR	SOL	TP
**Spasticity**																						
Turna et al. 2018*																				× 100	× 100	× 100
Zeuner et al. 2016	× 30–60	× 25	× 40–60	× 30–100	× 50	× 40–100	× 20–60	× 15–50	× 20–50	× 30–50	× 30–100	× 30–100	× 10–30	× 10–20	× 40–80							
Picelli et al. 2014									× 50	× 50	× 83	× 50										
Ploumis et al. 2013*	× 63-95			× 63-95		× 63-95	× 63-95		× 63-95	× 63-95	× 63-95	× 63-95	× 63-95			× 63-95	× 63-95	× 63-95	× 63-95	× 63-95	× 63-95	
Picelli et al. 2012																				× 200 (100 in each head)		

Mayer et al. 2008	× 60	× 30

Clarifications

1) The dose/dose range are reported in units (U) of Onabotulinum A . In case of different BoNT types, a conversion ratio of 3:1 (Abobotulinum A:Onabotulinum A) was used to calculate the equivalent amounts, according to Reference 50 of the main manuscript.

2)In the studies by Turna et al. and Ploumis et al., the indicated dose per muscle is an approximation and not the exact dose or dose range, because these were not reported by the authors. The dose per muscle was estimated based on the total dose. In the study by Turna et al. it was assumed that all three muscles were injected, whereas in the study by Ploumis et al. it was assumed that 4-6 muscles were injected in each patient, according to the table with individual patient data in their manuscript.

Table S2. Risk of bias assessment across all domains using the revised Cochrane risk-of-bias tool (RoB 2 tool) for randomized trials and the Newcastle-Ottawa scale for one non-randomized study.

**RoB2 tool (parallel-group)**	**Randomization processs**	**Effect of assignment to intervention**	**Missing outcome data**	**Measurement of the outcome**	**Selection of the reported result**	**Overall risk of bias**
**Picelli et al. (2014)**	***Low***	*Low*	*Low*	*Low*	*Low*	*Low*
Ploumis et al. (2013)	*Low*	*Low*	*Low*	*Low*	*Low*	*Low*
Picelli et al. (2012)	*Low*	*Low*	*Low*	*Low*	*Low*	*Low*
Mayer et al. (2008)	*Some concerns*	*Low*	*Low*	*Low*	*Low*	*Some concerns*
Zeuner et al. (2016) ***RoB2 tool (crossover trial): Low risk of bias from carryover effects**	*Some concerns*	*Low*	*Low*	*Low*	*Low*	*Some concerns*

**Newcastle-Ottawa scale**	**Representativeness of the exposed cohort**	**Selection of the non exposed cohort**	**Ascertainment of exposure**	**Outcome present at start of study**	**Comparability of cohorts (design or analysis)**	**Assessment of outcome**	**Was follow-up long enough for outcomes to occur**	**Adequacy of follow up of cohorts**
Turna et al. (2018)	*	*	*	*	**	*	*	*

Table S3. Certainty of evidence rating according to the GRADE guidelines. EMG: electromyography, ES: electrical stimulation, GRADE: Grading of Recommendations Assessment, Development and Evaluation, US: ultrasound, MNP: manual needle placement *As there were published studies with no difference (negative) results and we also checked for unpublished studies we decided by default not to downgrade any study for publication bias. .

Comparison	Study Limitations	Imprecision	Inconsistency	Indirectness	Publication Bias*	GRADE
US vs MNP	No downgrade	No downgrade	No downgrade	No downgrade	No downgrade	HIGH
EMG vs MNP	No downgrade	No downgrade	No downgrade	No downgrade	No downgrade	HIGH
EMG vs US	Downgrade	Downgrade	No downgrade	No downgrade	No downgrade	LOW
ES vs MNP	No downgrade	No downgrade	No downgrade	No downgrade	No downgrade	HIGH
ES vs US	No downgrade	No downgrade	No downgrade	No downgrade	No downgrade	HIGH
ES vs EMG	Downgrade	No downgrade	No downgrade	No downgrade	No downgrade	MODERATE

Table S4: Inconsistency test with the node-split method (local approach). For each comparison the p-values are provided and in all cases, they are greater than 0.05, meaning that there is no conflict between direct and indirect evidence. EMG: electromyography, ES: electrical stimulation, MNP: manual needle placement, ultrasound, 95% CrI: Credible interval

Comparison	p-value	Mean Difference (95% CrI)
**EMG vs ES**	0.989625	–
direct	–	0.33 (-0.038, 0.69)
indirect	–	0.32 (-0.59,1.4)
network	–	0.33 (0.065, 0.63)
**EMG vs MNP**	0.834475	–
direct	–	-0.36 (-1.1, 0.48)
indirect	–	-0.48 (-1.1, 0.12)
network	–	-0.48 (-0.95, 0.012)
**EMG vs US**	0.961450	–
direct	–	0.22 (-5.7, 6.6)
indirect	–	0.33 (0.12, 0.60)
network	–	0.33 (0.037, 0.71)
**ES vs MNP**	0.879600	–
direct	–	-0.81 (-1.7, 0.081)
indirect	–	-0.70 (-2.3, 0.89)
network	–	-0.81 (-1.2,-0.34)

**ES vs US**	0.835150	–
direct	–	0.00051 (-0.28, 0.38)
indirect	–	0.55 (-6.5, 8.3)
network	–	0.00048 (-0.17, 0.24)
**US vs MNP**	0.807825	–
direct	–	0.95 (0.029, 1.9)
indirect	–	0.75 (-1.3, 2.7)
network	–	0.82 (0.35, 1.3)

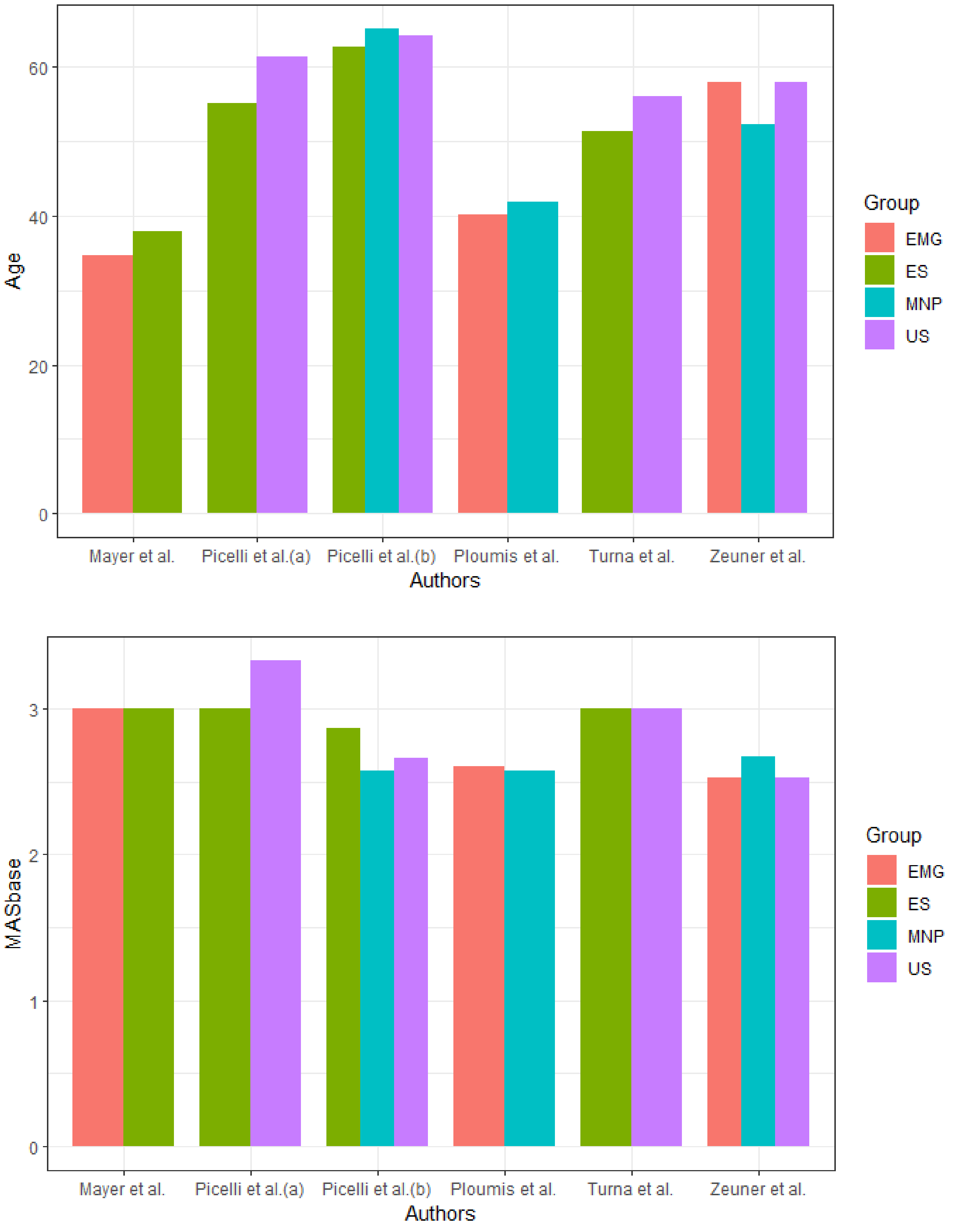

Supplementary Figure S1: Bar plots showing baseline characteristics in each group across studies. Upper graph: Mean age and lower graph: MAS at baseline **Abbreviations:** EMG: electromyography, ES: electrical stimulation, MASbase: Modified Ashworth Scale at baseline, MNP: manual needle placement, US: ultrasound

Appendix

Sensitivity Analysis

In the following section the numerical estimates and the plots represent the output of the MetaInsight software when we excluded the mentioned study each time. The order of the tables and plots is the following:

- *Forest plot (Bayesian framework)*
- *Ranking Table with SUCRA values*
- *Litmus Rank-O-Gram*
- *SUCRA radial plot*
- *Deviance report (NMA/UME residual deviance plot, stem plot, leverage plot)*

Appendix

- *Gelman convergence assessment plot*
- *Forest plot (Frequentist framework)*
- *League table (Frequentist framework)*

(1) Exlusion of the study by Ploumis et al.

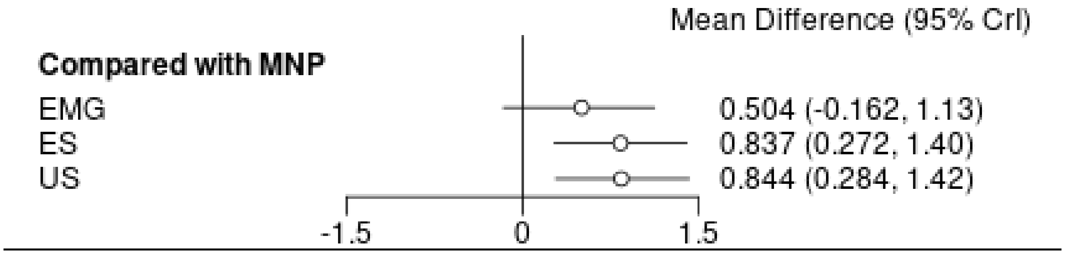

Treatment	Rank 1	Rank 2	Rank 3	Rank 4	SUCRA
US	0.51	0.46	0.02	0.00	82.89
ES	0.47	0.51	0.02	0.00	81.51
EMG	0.02	0.02	0.90	0.06	33.35
MNP	0.00	0.00	0.06	0.94	2.26

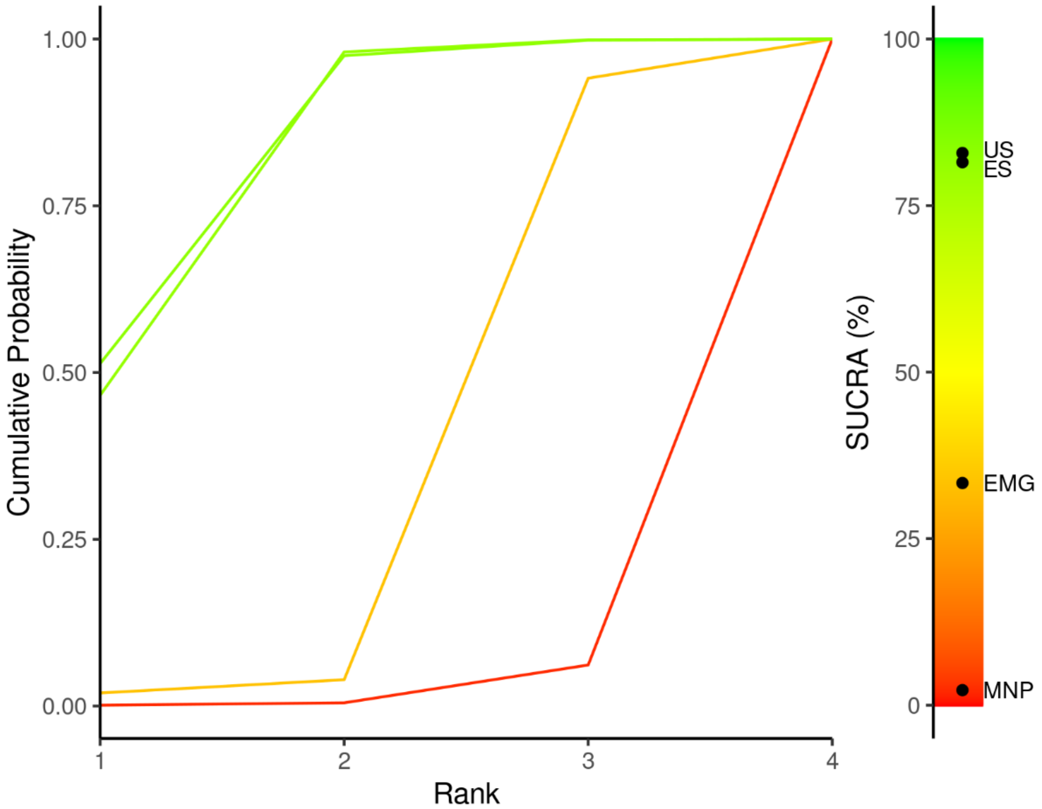

1.00
0.75
0.50
0.25
0.00
Cumulative Probability
1
2
3
4
Rank
100
75
50
25
0
SUCRA (%)
US
ES
EMG
MNP

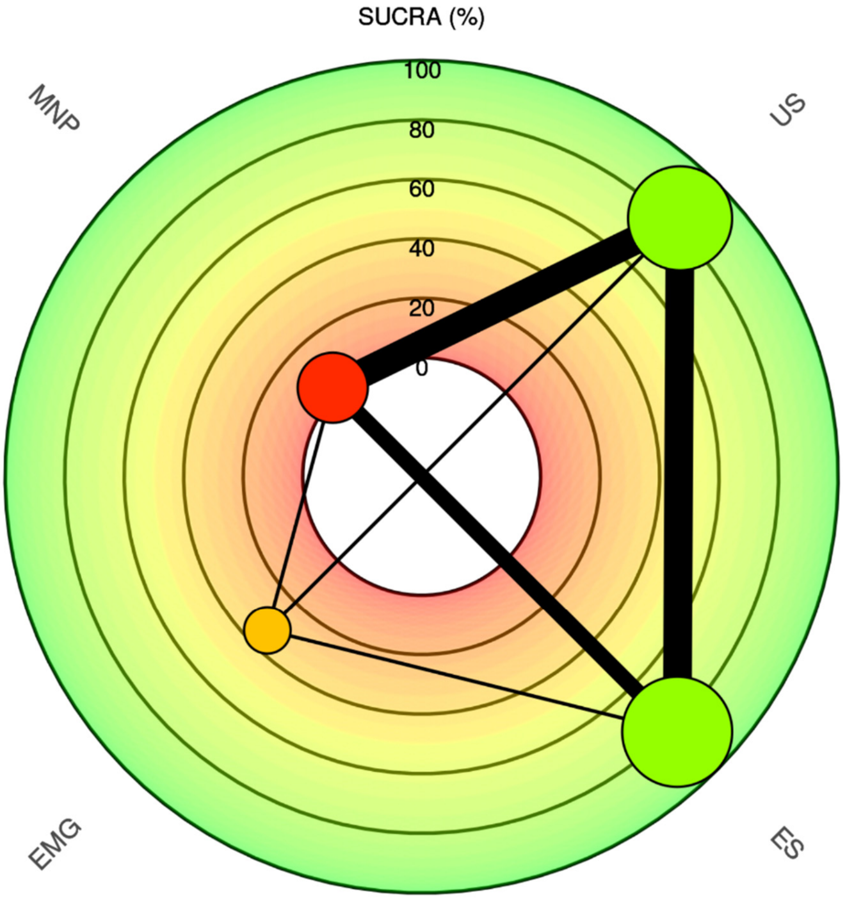

SUCRA (%)
100
80
60
40
20
0
MNP
US
EMG
ES

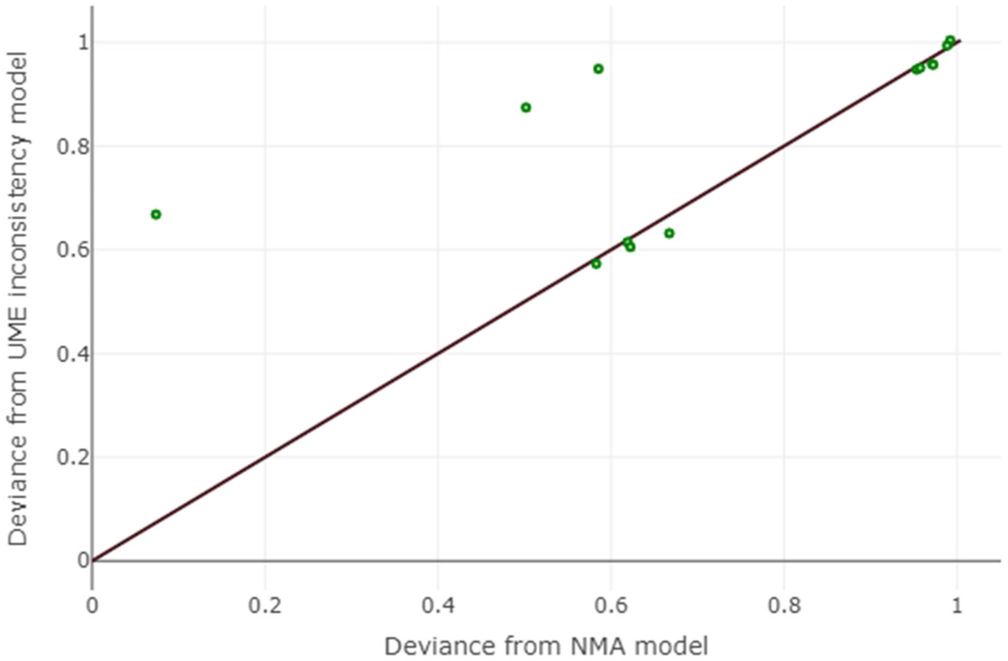
Deviance from UME inconsistency model
Deviance from NMA model
0
0.2
0.4
0.6
0.8
1

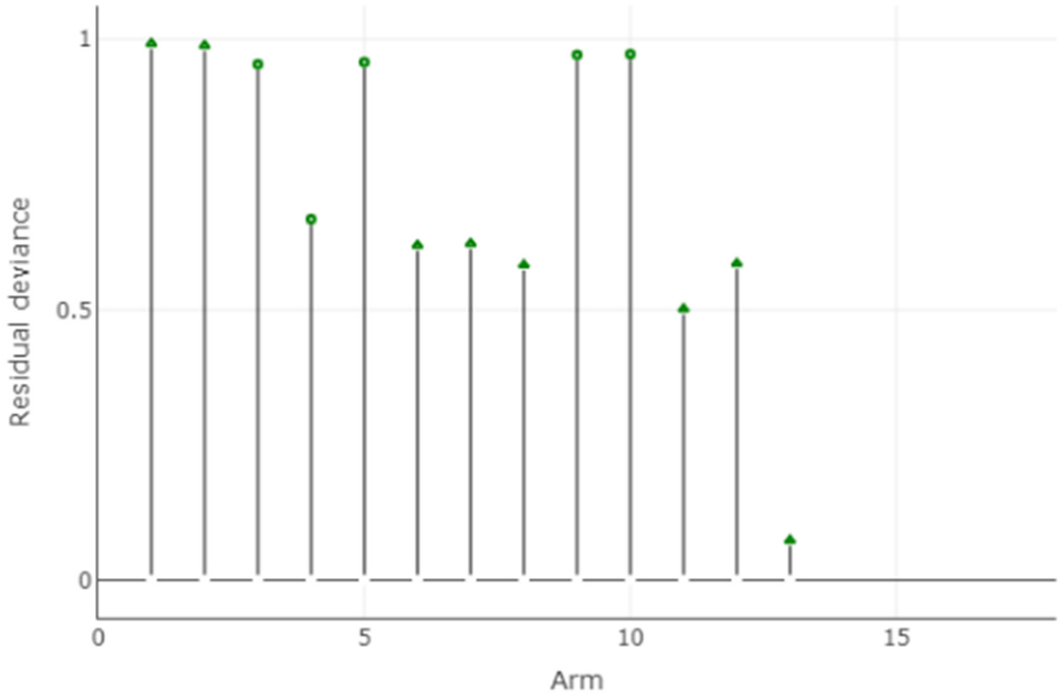
Residual deviance
Arm
0
0.5
1
5
10
15

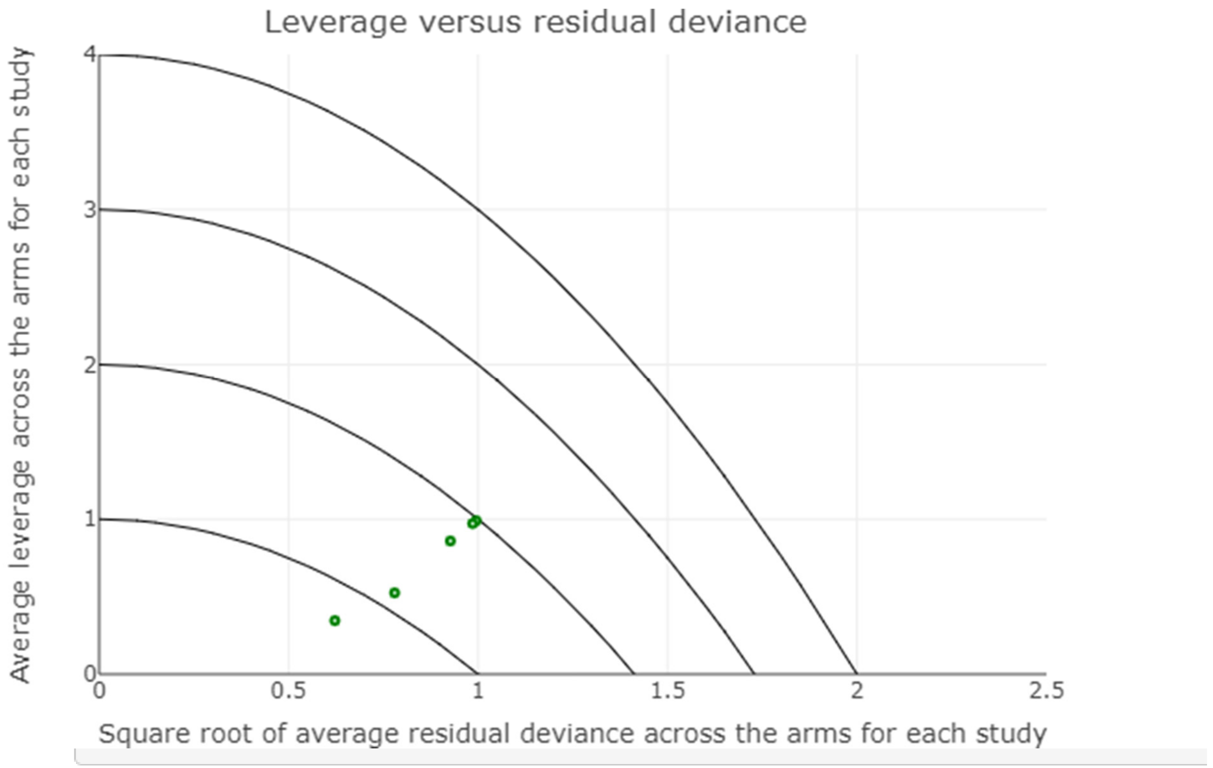

Gelman convergence assessment plot with studies excluded

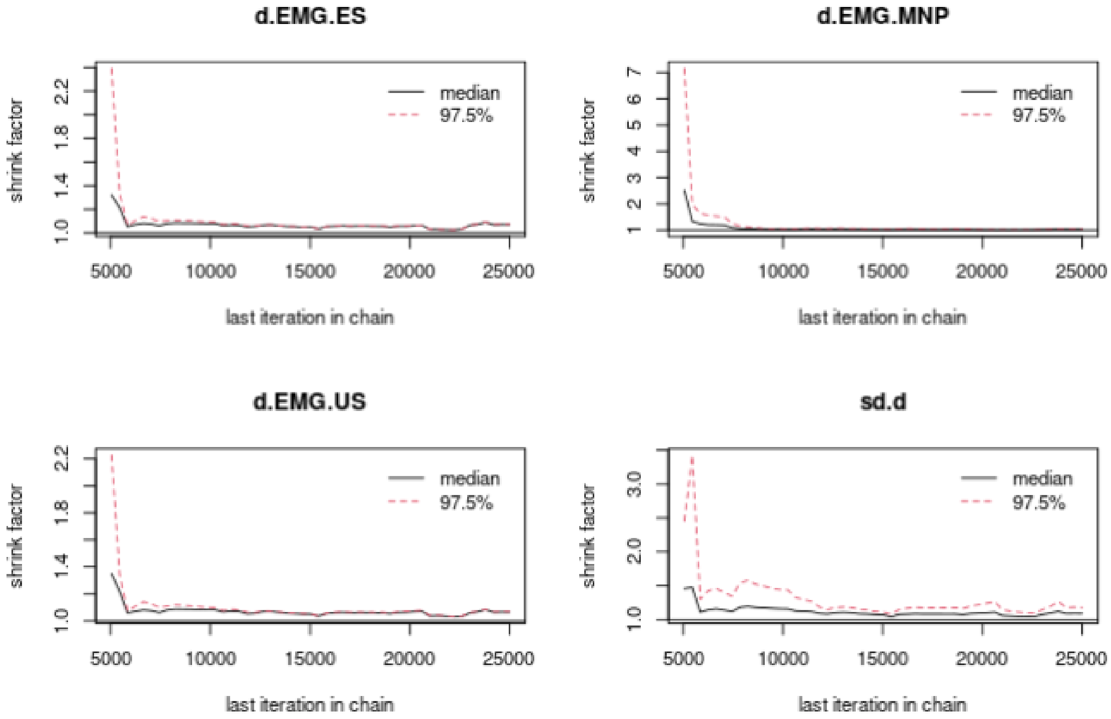

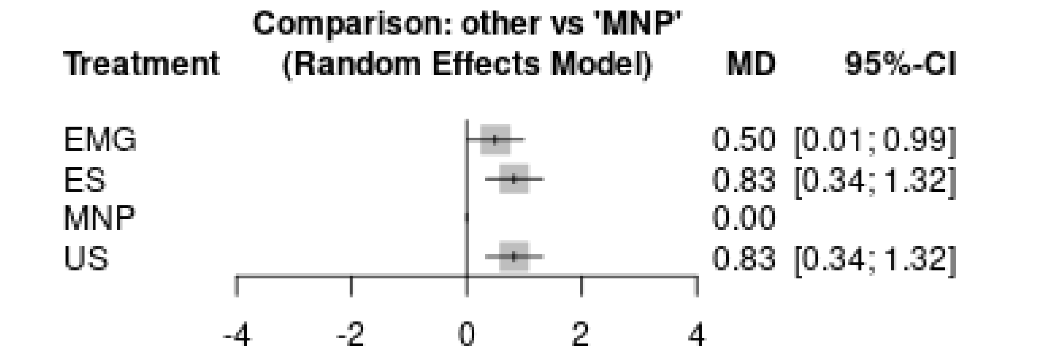

US	0.00 [0.00; 0.00]	0.47 [-7.24; 8.18]	0.88 [0.36; 1.40]
0.00 [0.00; 0.00]	ES	0.33 [0.32; 0.34]	0.80 [0.29; 1.32]
0.33 [0.32; 0.34]	0.33 [0.32; 0.34]	EMG	-0.19 [-4.01; 3.63]
0.83 [0.34; 1.32]	0.83 [0.34; 1.32]	0.50 [0.01; 0.99]	MNP

(2) <u>Exlusion of the study by Turna et al.</u>

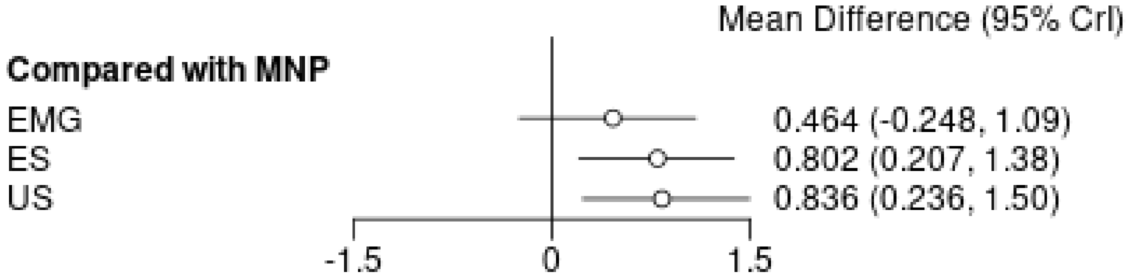

Treatment	Rank 1	Rank 2	Rank 3	Rank 4	SUCRA
US	0.55	0.38	0.06	0.00	82.77
ES	0.41	0.54	0.04	0.00	78.62
EMG	0.03	0.07	0.82	0.07	35.56
MNP	0.00	0.01	0.07	0.92	3.05

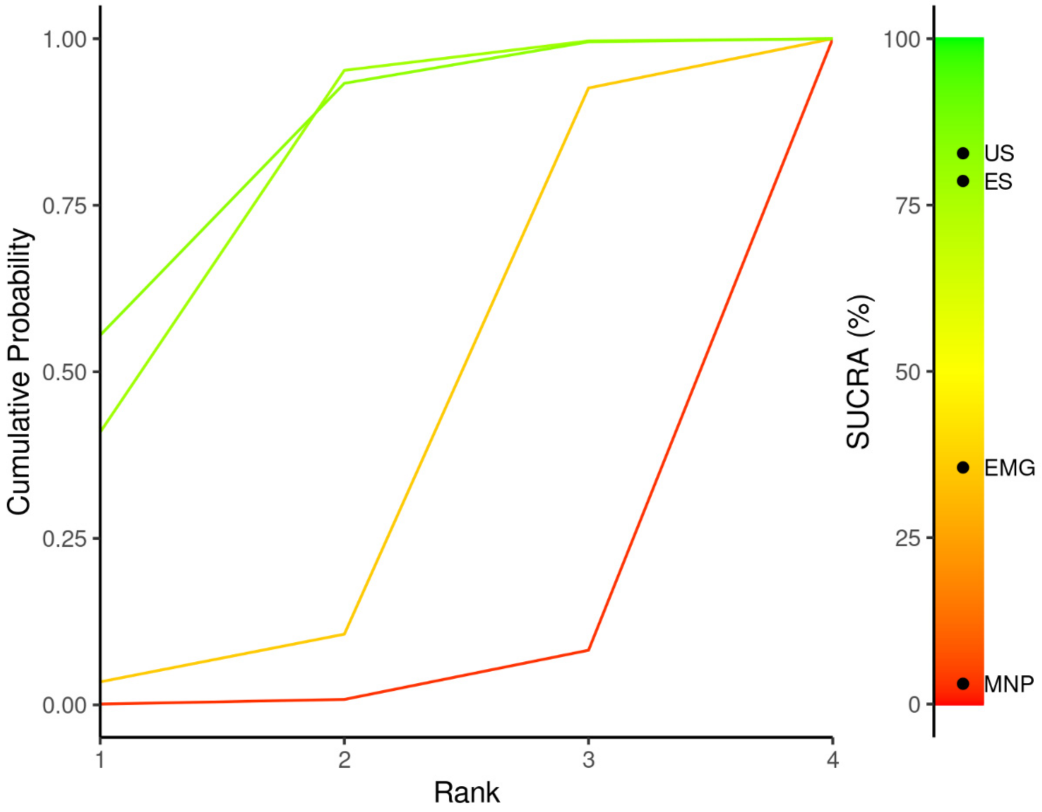
Cumulative Probability
1.00
0.75
0.50
0.25
0.00
Rank
1
2
3
4
SUCRA (%)
100
75
50
25
0
US
ES
EMG
MNP

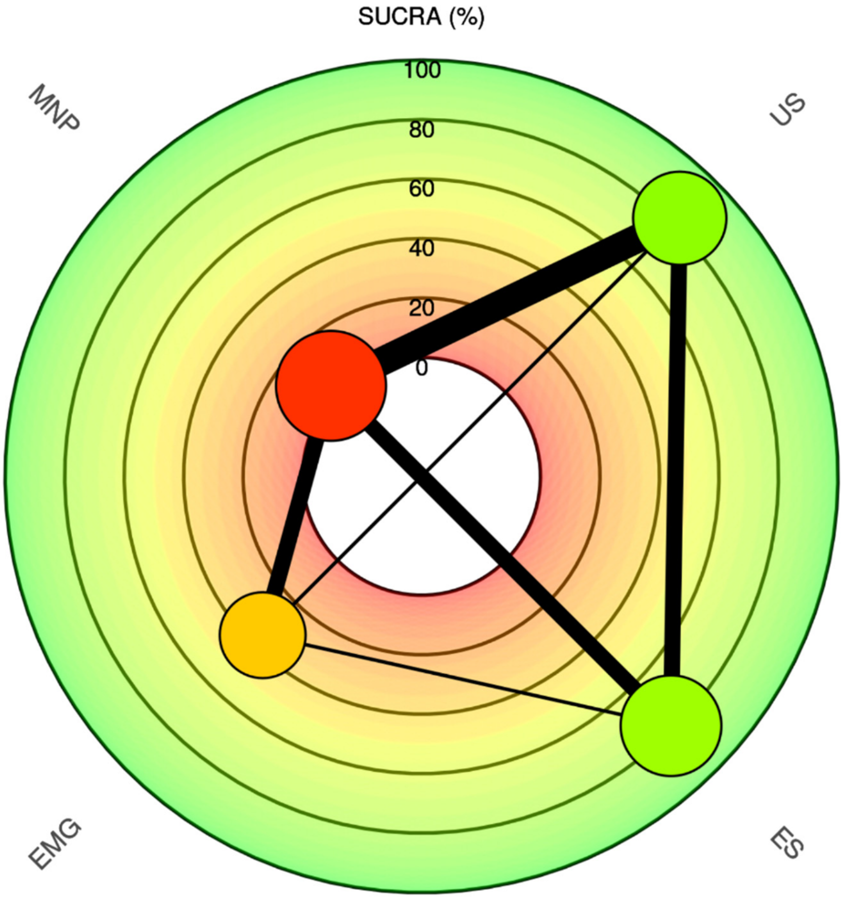
SUCRA (%)
100
80
60
40
20
0
MNP
US
EMG
ES

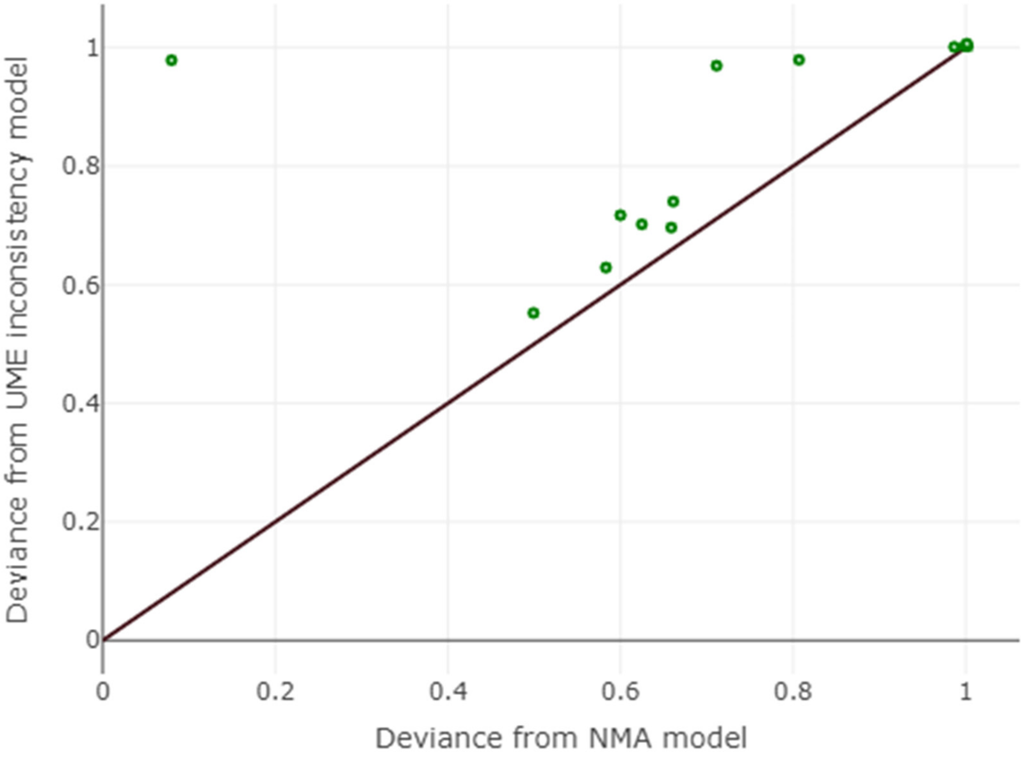

Deviance from UME inconsistency model
0
0.2
0.4
0.6
0.8
1
0
0.2
0.4
0.6
0.8
1
Deviance from NMA model

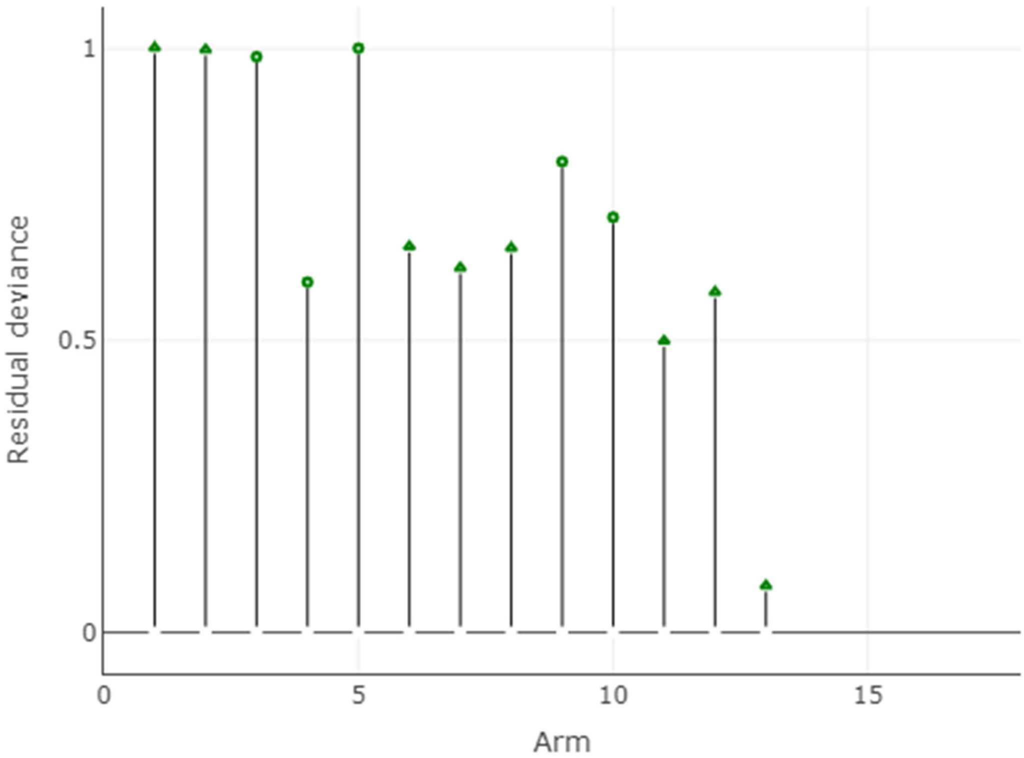

Residual deviance
0
0.5
1
0
5
10
15
Arm

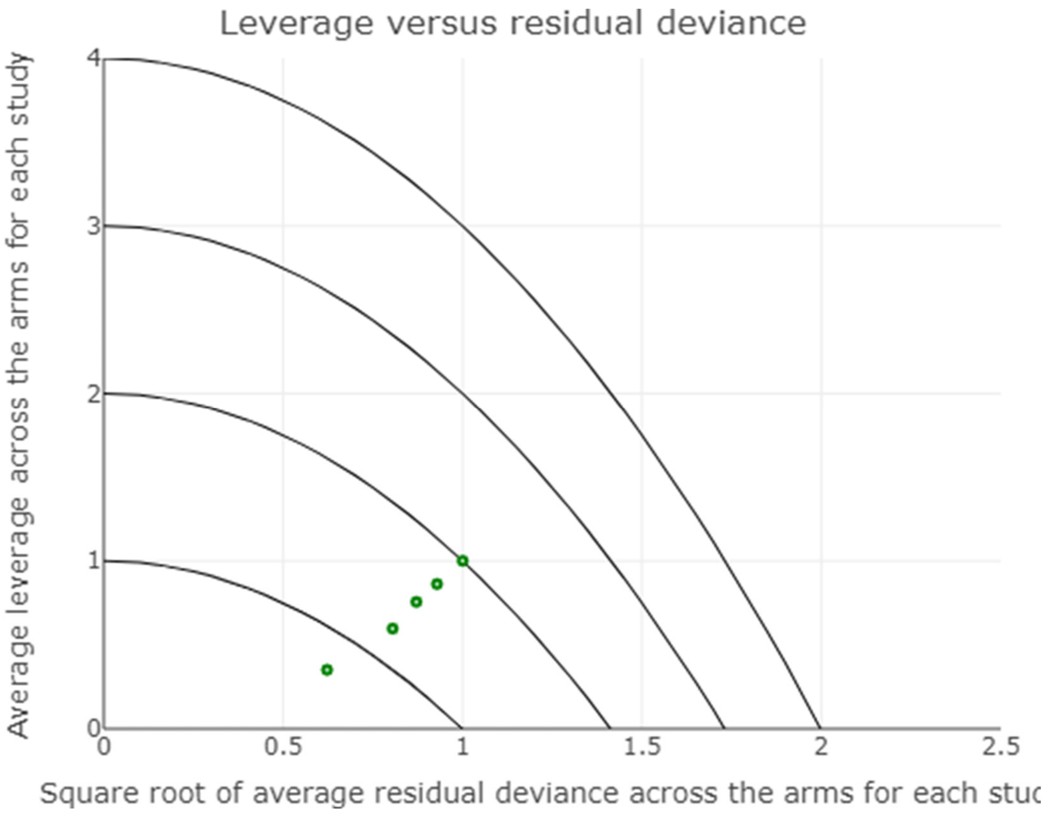
Leverage versus residual deviance
Average leverage across the arms for each study
Square root of average residual deviance across the arms for each stuc
0
0.5
1
1.5
2
2.5
1
2
3
4

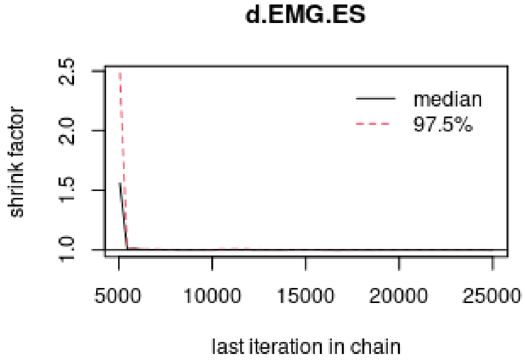
d.EMG.ES
shrink factor
median
97.5%
last iteration in chain

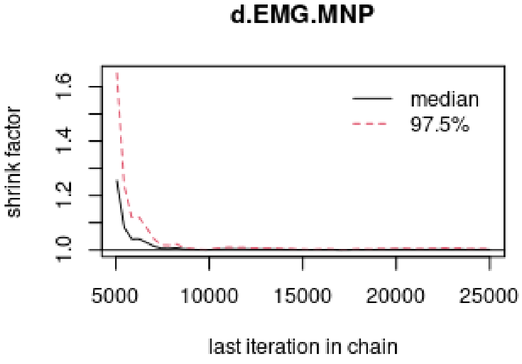
d.EMG.MNP
shrink factor
median
97.5%
last iteration in chain

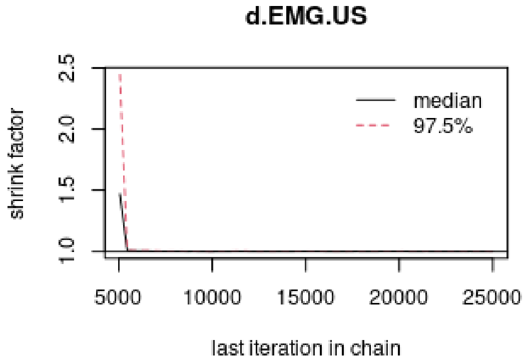
d.EMG.US
shrink factor
median
97.5%
last iteration in chain

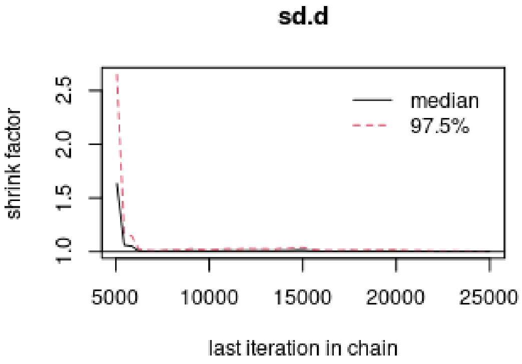
sd.d
shrink factor
median
97.5%
last iteration in chain

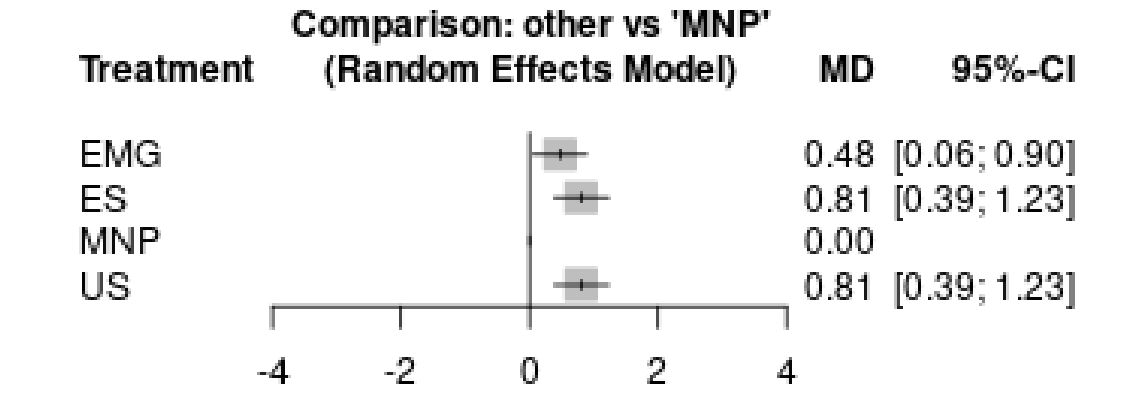

US	0.00 [-0.01; 0.01]	0.47 [-7.24; 8.18]	0.88 [0.36; 1.40]
0.00 [-0.01; 0.01]	ES	0.33 [0.32; 0.34]	0.80 [0.29; 1.32]
0.33 [0.32; 0.34]	0.33 [0.32; 0.34]	EMG	0.39 [-0.41; 1.20]
0.81 [0.39; 1.23]	0.81 [0.39; 1.23]	0.48 [0.06; 0.90]	MNP

(3) <u>Exlusion of the study by Zeuner et al.</u>

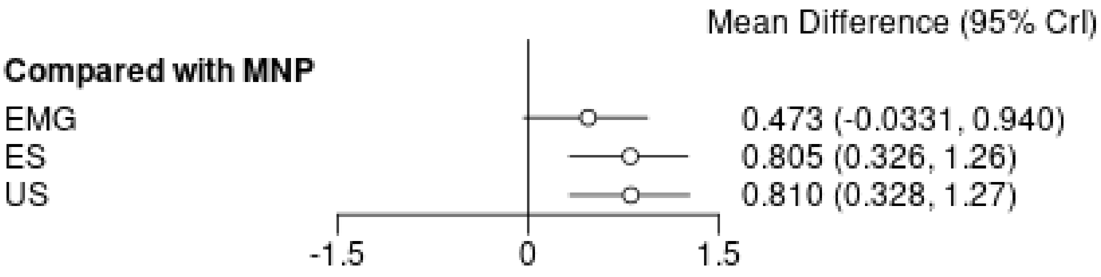

Treatment	Rank 1	Rank 2	Rank 3	Rank 4	SUCRA
US	0.52	0.47	0.01	0.00	83.60
ES	0.47	0.52	0.01	0.00	82.20
EMG	0.01	0.01	0.95	0.03	33.08
MNP	0.00	0.00	0.03	0.97	1.12

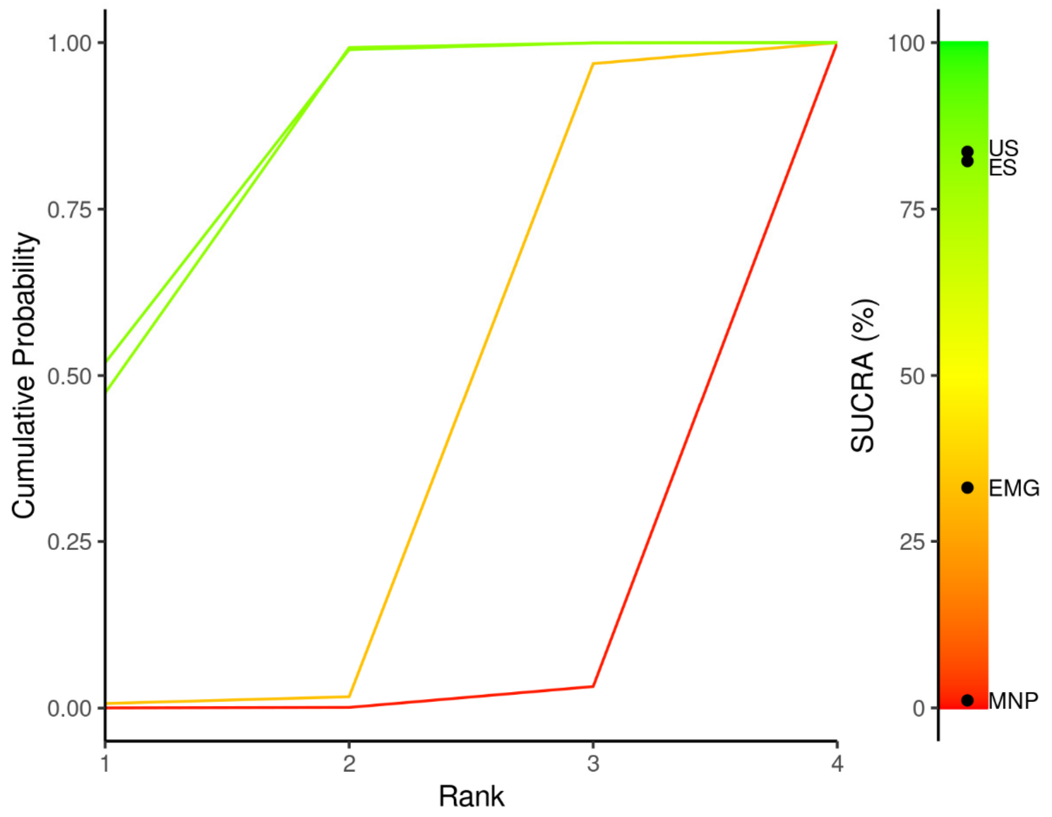
1.00
0.75
0.50
0.25
0.00
Cumulative Probability
1
2
3
4
Rank
100
75
50
25
0
SUCRA (%)
US
ES
EMG
MNP

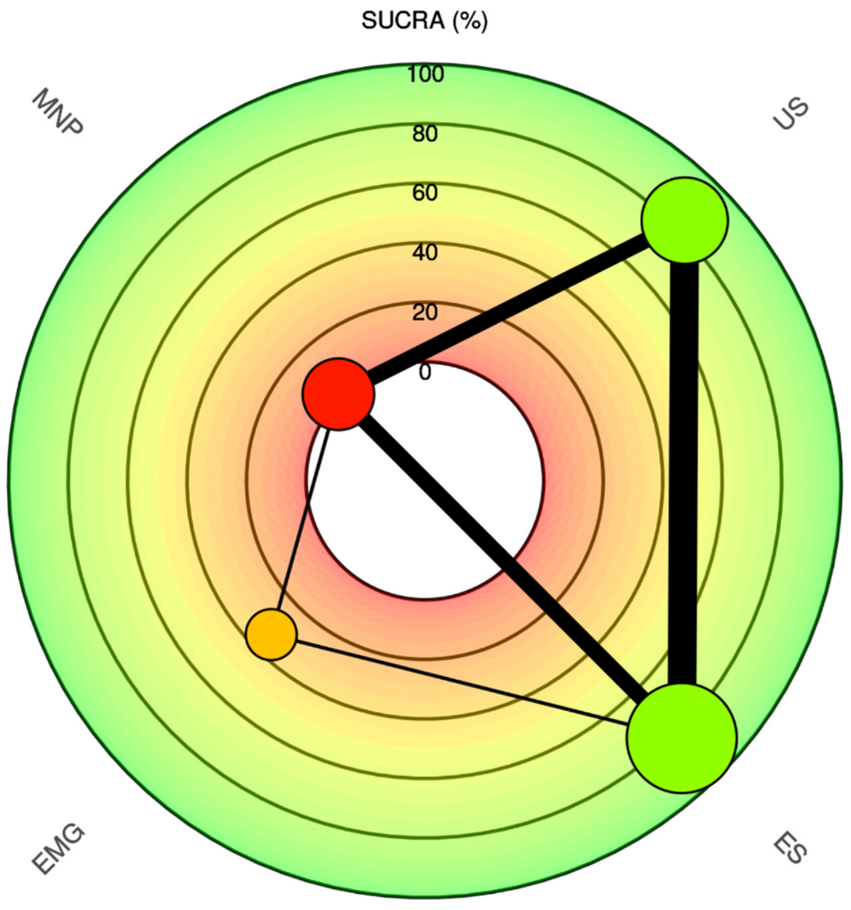
SUCRA (%)
100
80
60
40
20
0
MNP
US
EMG
ES

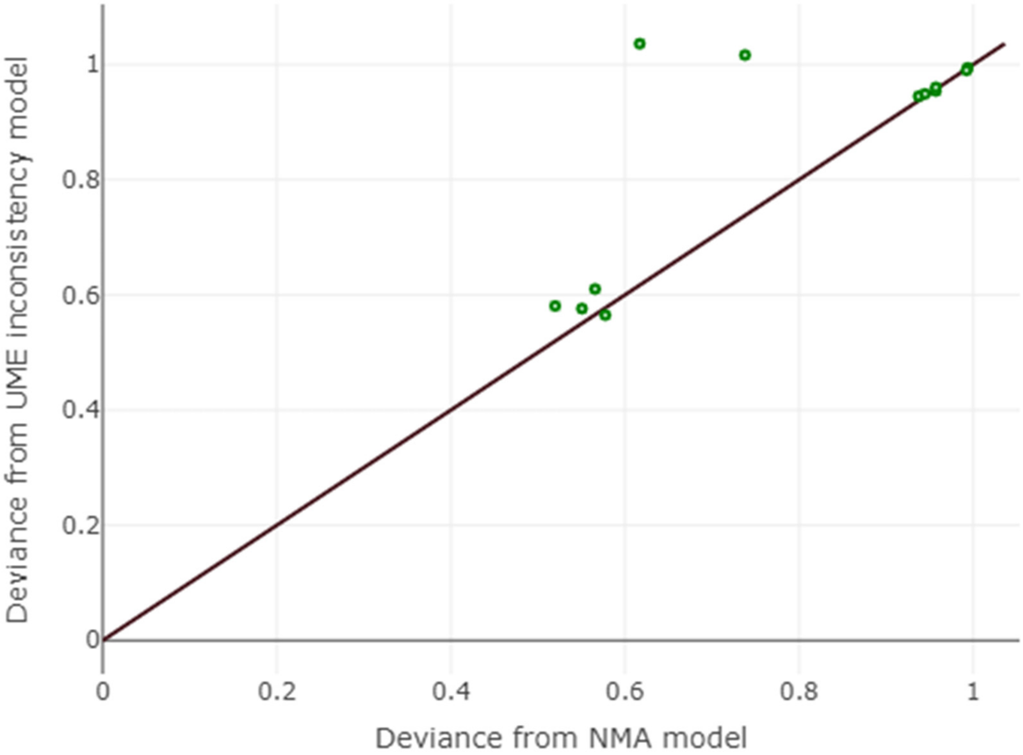

Deviance from UME inconsistency model
1
0.8
0.6
0.4
0.2
0
0
0.2
0.4
0.6
0.8
1
Deviance from NMA model

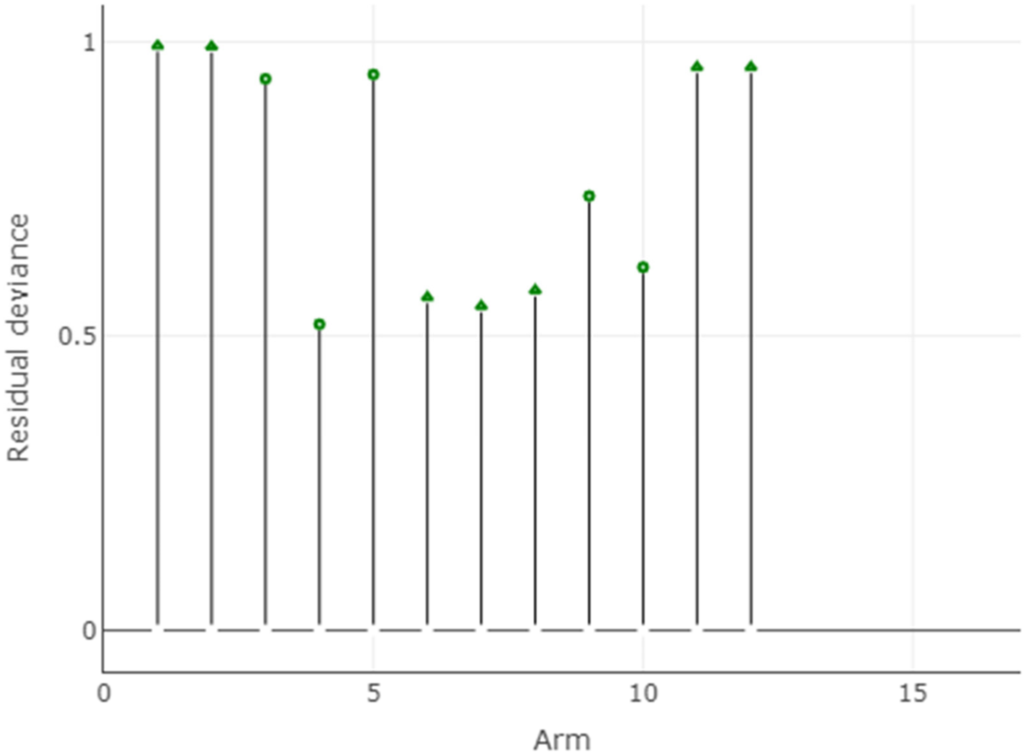

Residual deviance
1
0.5
0
0
5
10
15
Arm

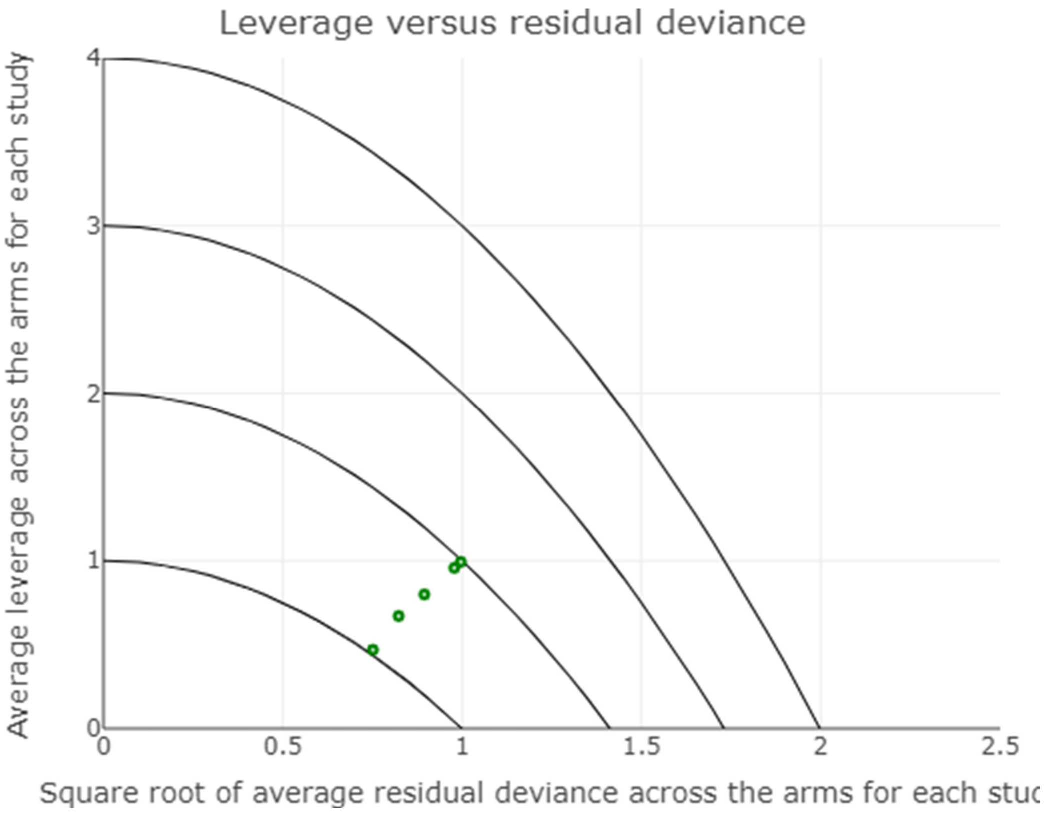
Leverage versus residual deviance
Average leverage across the arms for each study
Square root of average residual deviance across the arms for each stuc
0
1
2
3
4
0
0.5
1
1.5
2
2.5

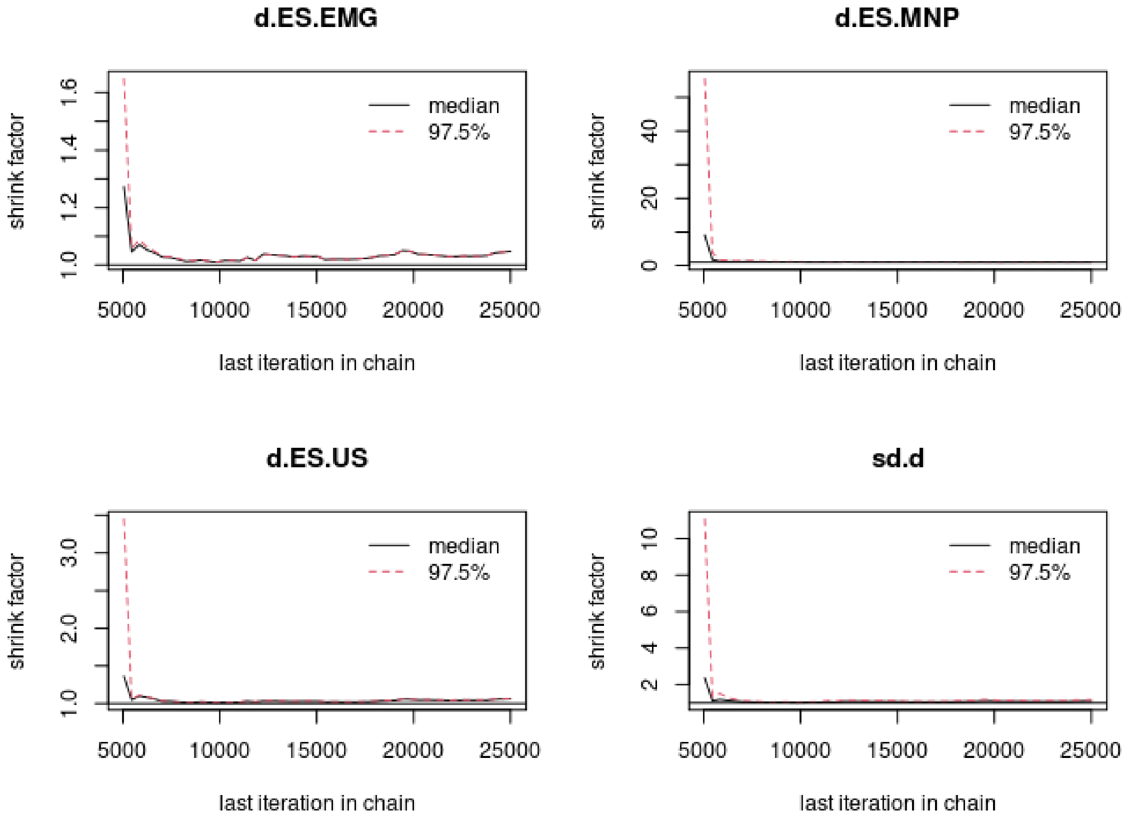
d.ES.EMG
d.ES.MNP
d.ES.US
sd.d
shrink factor
median
97.5%
last iteration in chain
5000
10000
15000
20000
25000

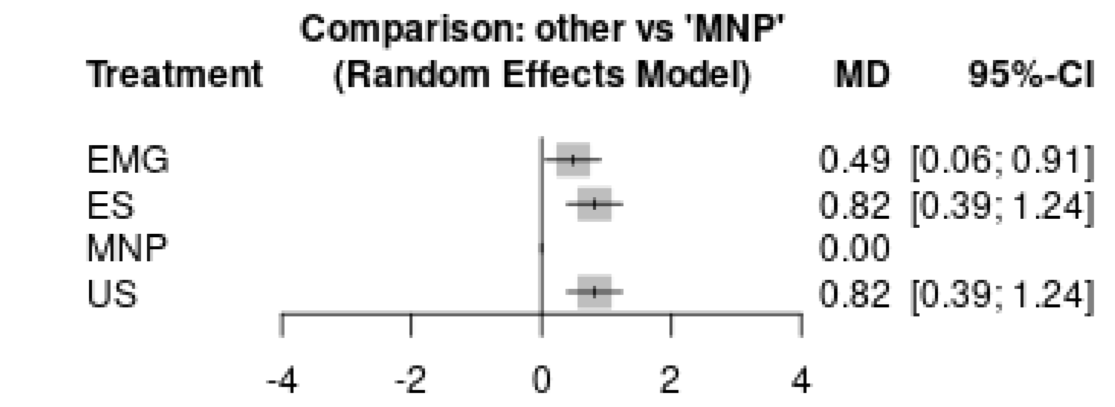

US	0.00 [0.00; 0.00]	.	0.88 [0.36; 1.41]
0.00 [0.00; 0.00]	ES	0.33 [0.32; 0.34]	0.80 [0.29; 1.32]
0.33 [0.32; 0.34]	0.33 [0.32; 0.34]	EMG	0.42 [-0.40; 1.24]
0.82 [0.39; 1.24]	0.82 [0.39; 1.24]	0.49 [0.06; 0.91]	MNP

(4) Exlusion of the study by Mayer et al.

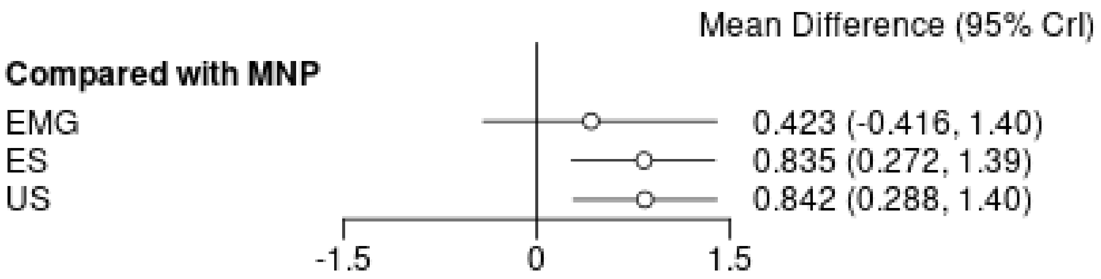

Treatment	Rank 1	Rank 2	Rank 3	Rank 4	SUCRA
US	0.42	0.48	0.10	0.00	77.25
ES	0.39	0.49	0.12	0.00	75.48
EMG	0.19	0.03	0.59	0.19	40.72
MNP	0.00	0.00	0.19	0.81	6.54

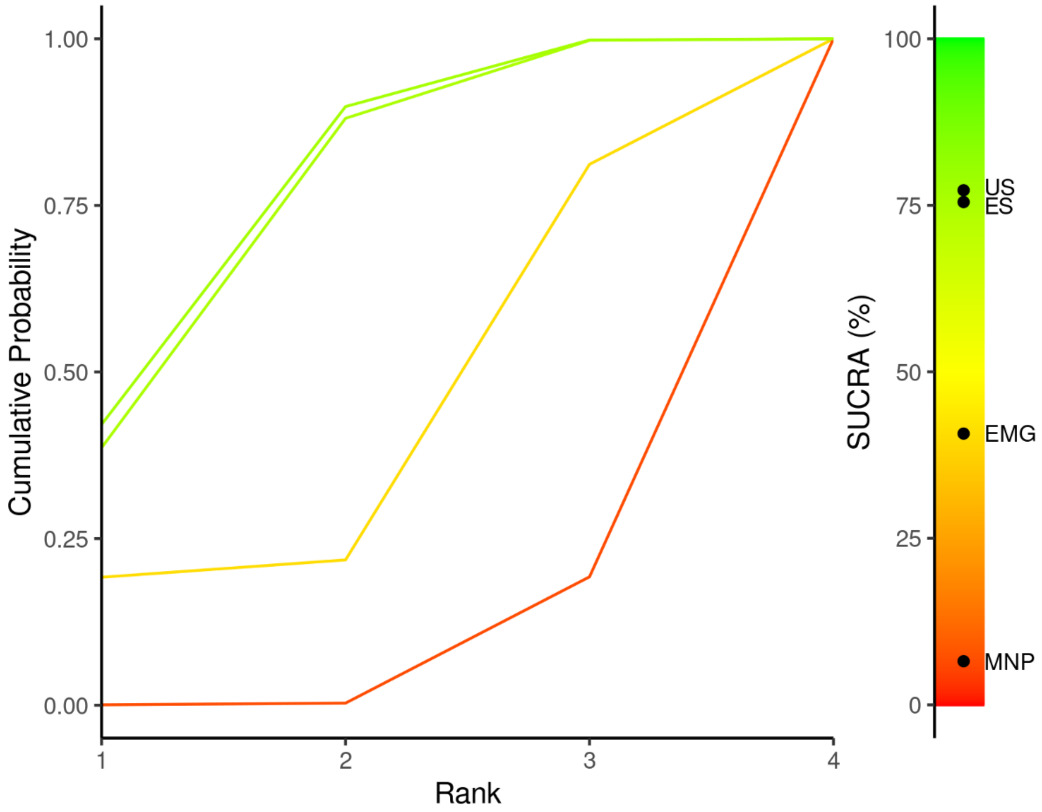

1.00
0.75
0.50
0.25
0.00
Cumulative Probability
1
2
3
4
Rank
100
75
50
25
0
SUCRA (%)
US
ES
EMG
MNP

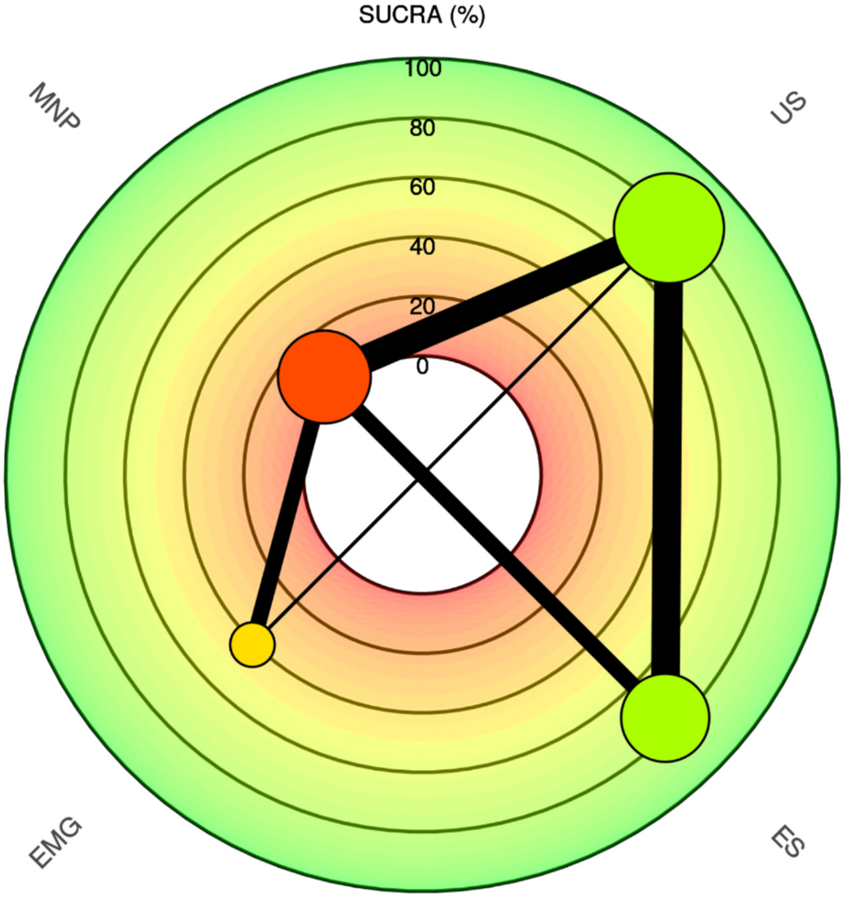

SUCRA (%)
100
80
60
40
20
0
MNP
US
EMG
ES

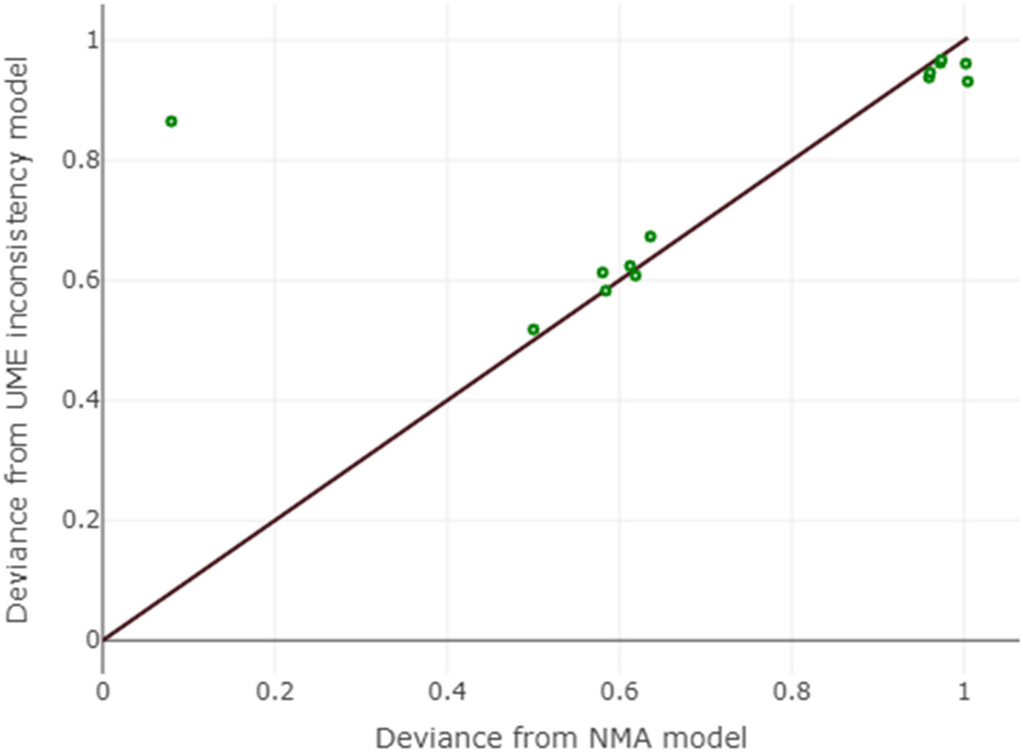
Deviance from UME inconsistency model
Deviance from NMA model
0
0.2
0.4
0.6
0.8
1

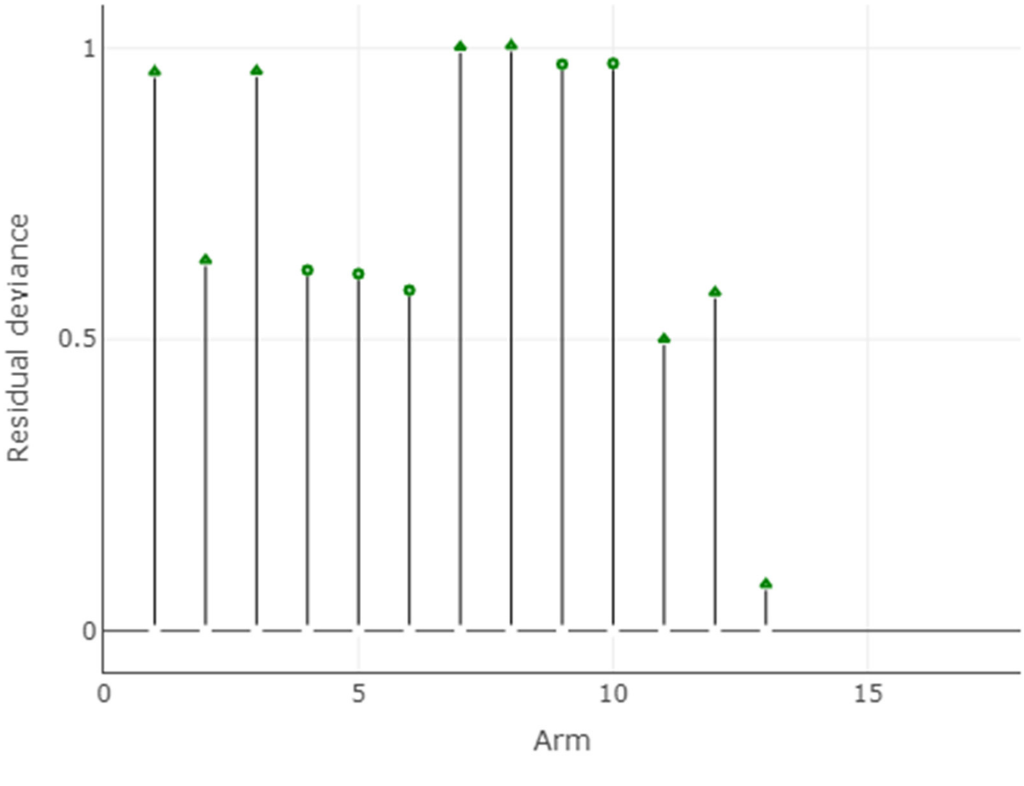
Residual deviance
Arm
0
0.5
1
5
10
15

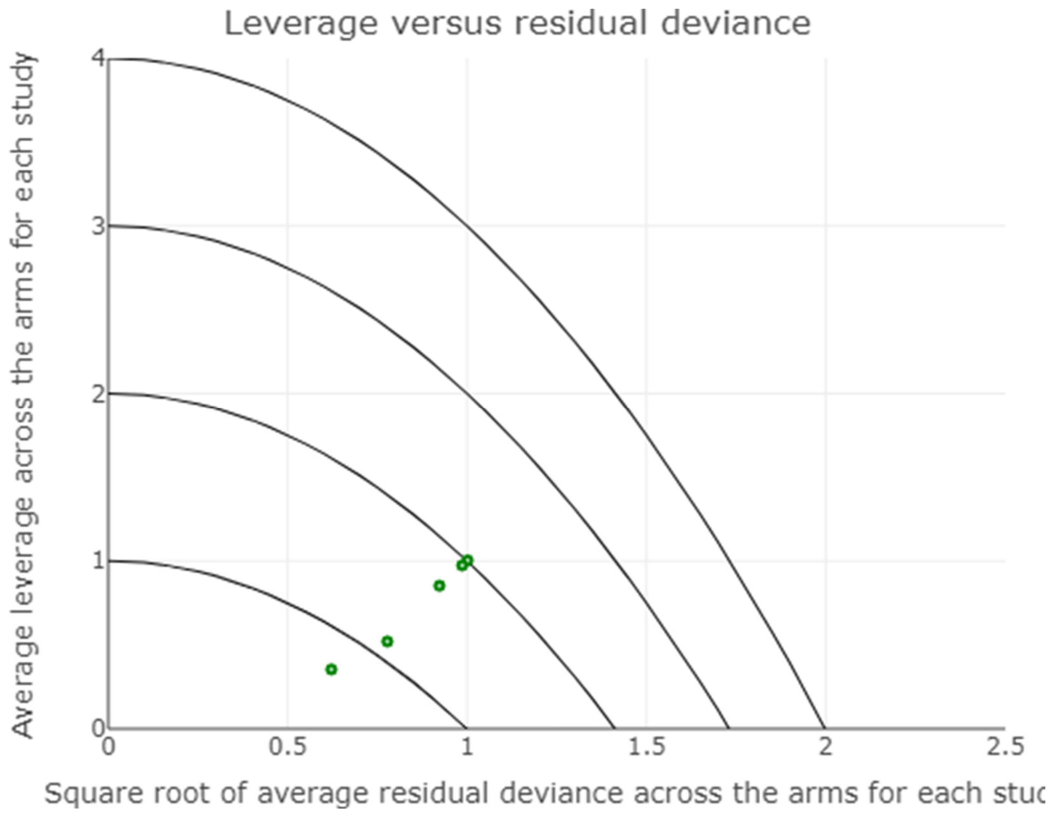

Leverage versus residual deviance
Average leverage across the arms for each study
Square root of average residual deviance across the arms for each stuc

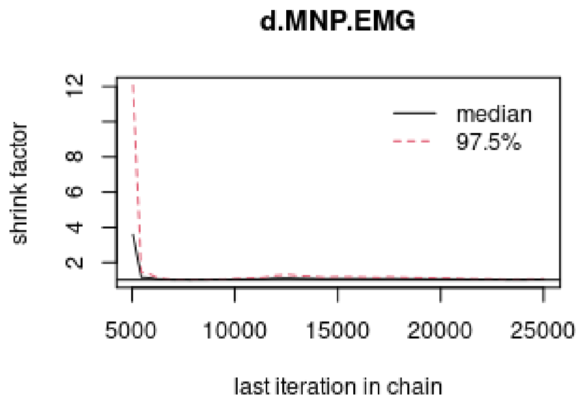

d.MNP.EMG
shrink factor
median
97.5%
last iteration in chain

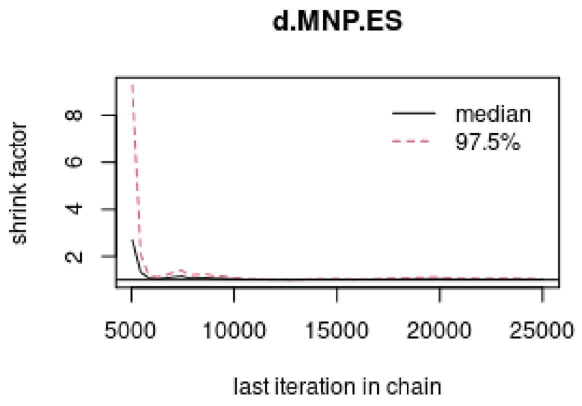

d.MNP.ES
shrink factor
median
97.5%
last iteration in chain

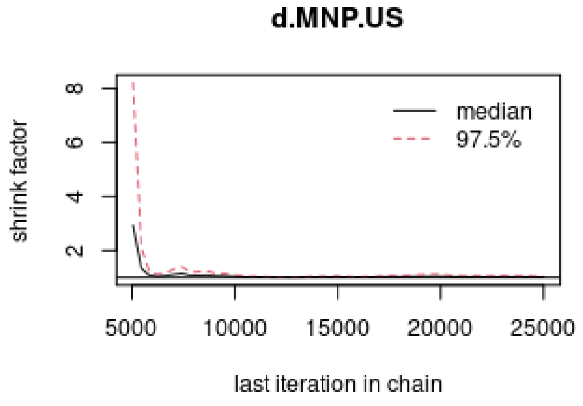

d.MNP.US
shrink factor
median
97.5%
last iteration in chain

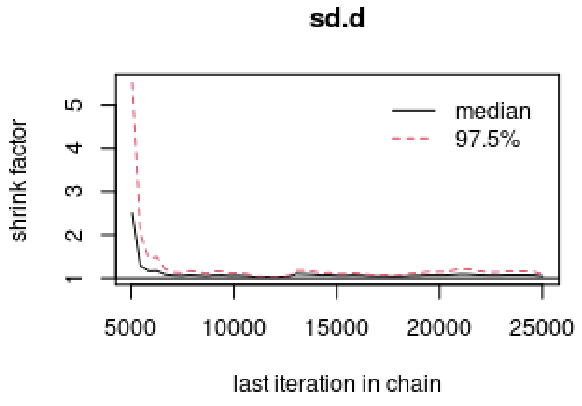

sd.d
shrink factor
median
97.5%
last iteration in chain

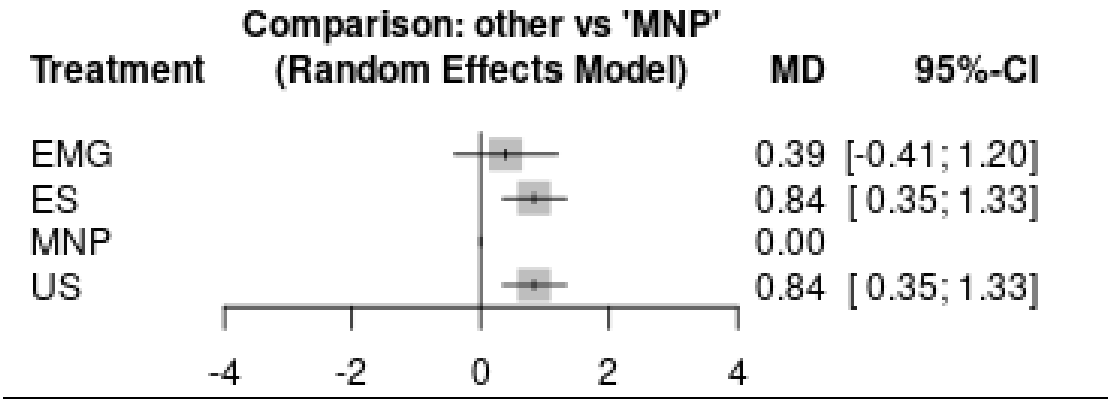

US	0.00 [0.00; 0.00]	0.47 [-7.24; 8.18]	0.88 [0.36; 1.40]
0.00 [0.00; 0.00]	ES	.	0.80 [0.29; 1.32]
0.45 [-0.50; 1.39]	0.45 [-0.50; 1.39]	EMG	0.39 [-0.41; 1.20]
0.84 [0.35; 1.33]	0.84 [0.35; 1.33]	0.39 [-0.41; 1.20]	MNP

(5) Exlusion of the study by Zeuner et al. (only the MNP arm)

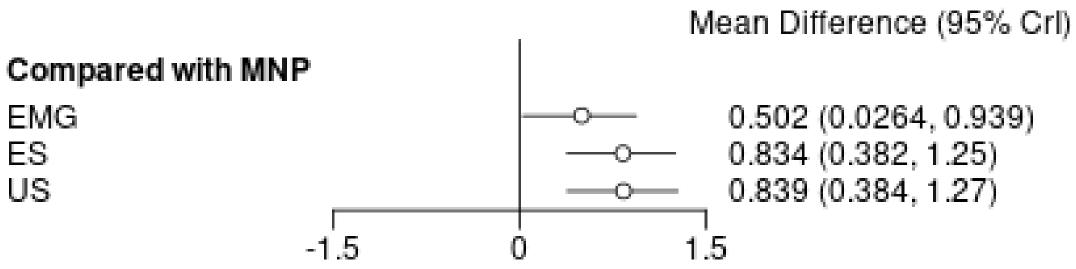

Treatment	Rank 1	Rank 2	Rank 3	Rank 4	SUCRA
US	0.52	0.47	0.01	0.00	83.62
ES	0.47	0.52	0.01	0.00	81.93
EMG	0.01	0.01	0.96	0.02	33.59
MNP	0.00	0.00	0.02	0.98	0.86

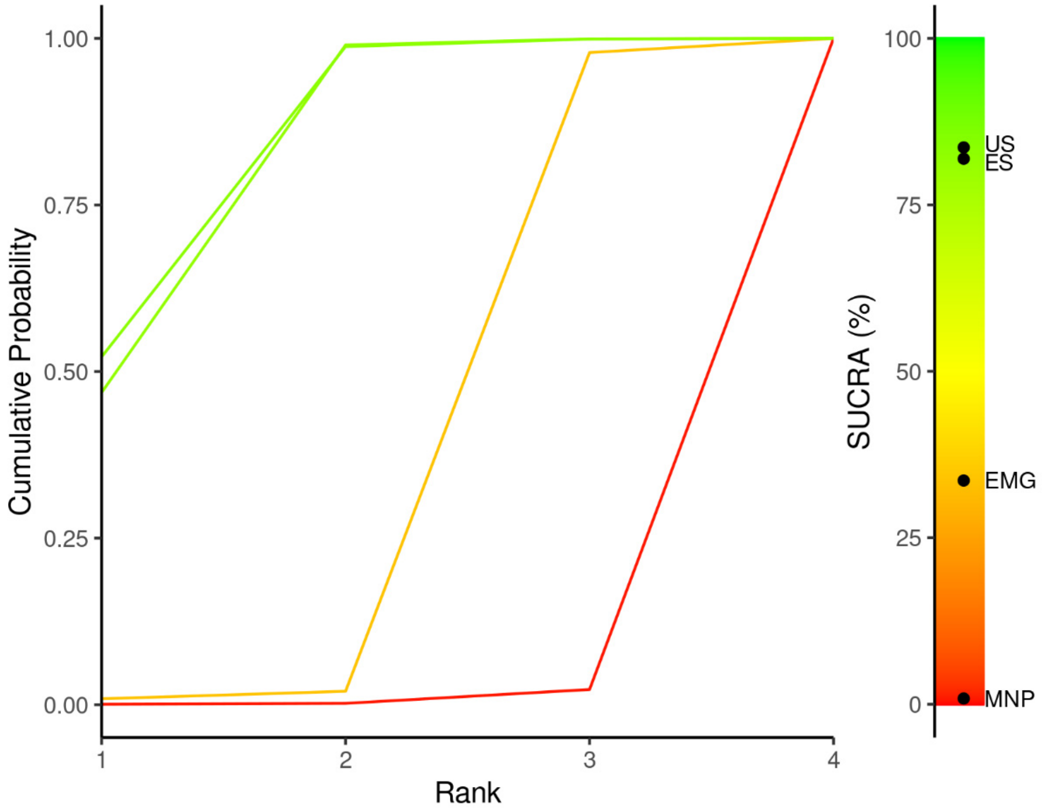

1.00
0.75
0.50
0.25
0.00
Cumulative Probability
1
2
3
4
Rank
100
75
50
25
0
SUCRA (%)
US
ES
EMG
MNP

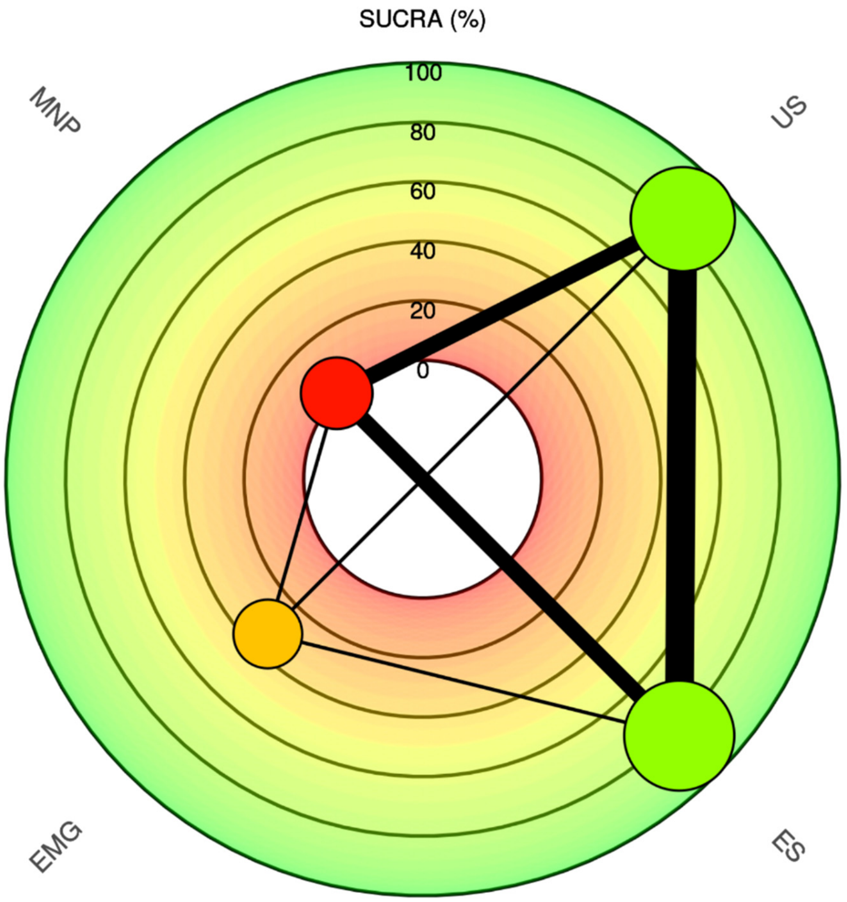

SUCRA (%)
100
80
60
40
20
0
MNP
US
EMG
ES

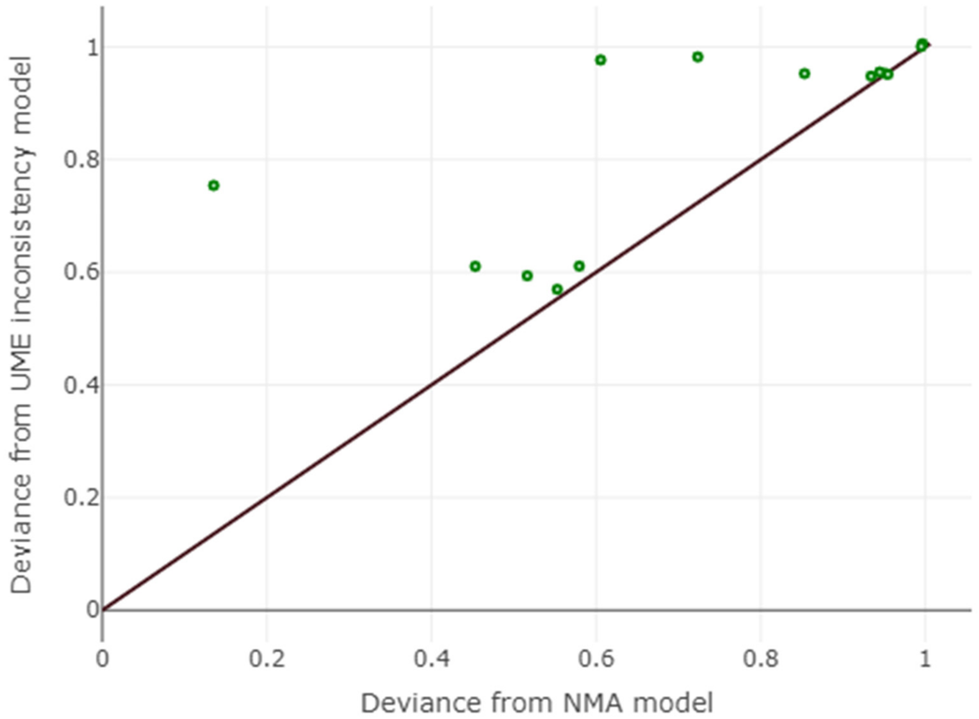

Deviance from UME inconsistency model
Deviance from NMA model
0
0.2
0.4
0.6
0.8
1

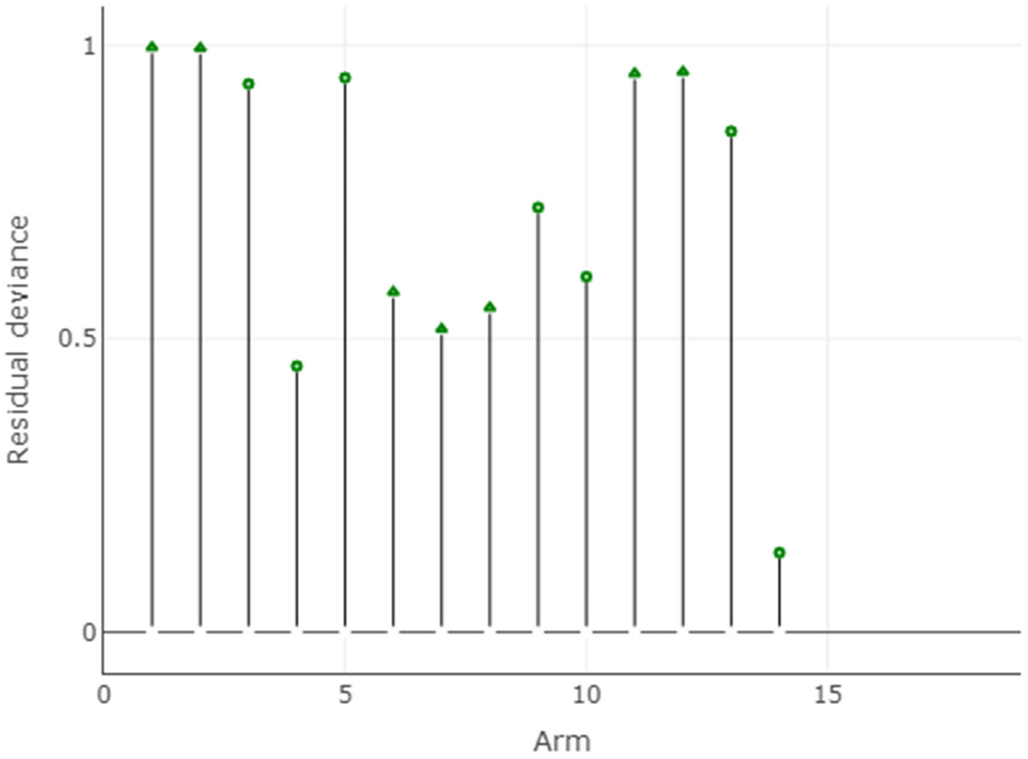

Residual deviance
Arm
0
0.5
1
5
10
15

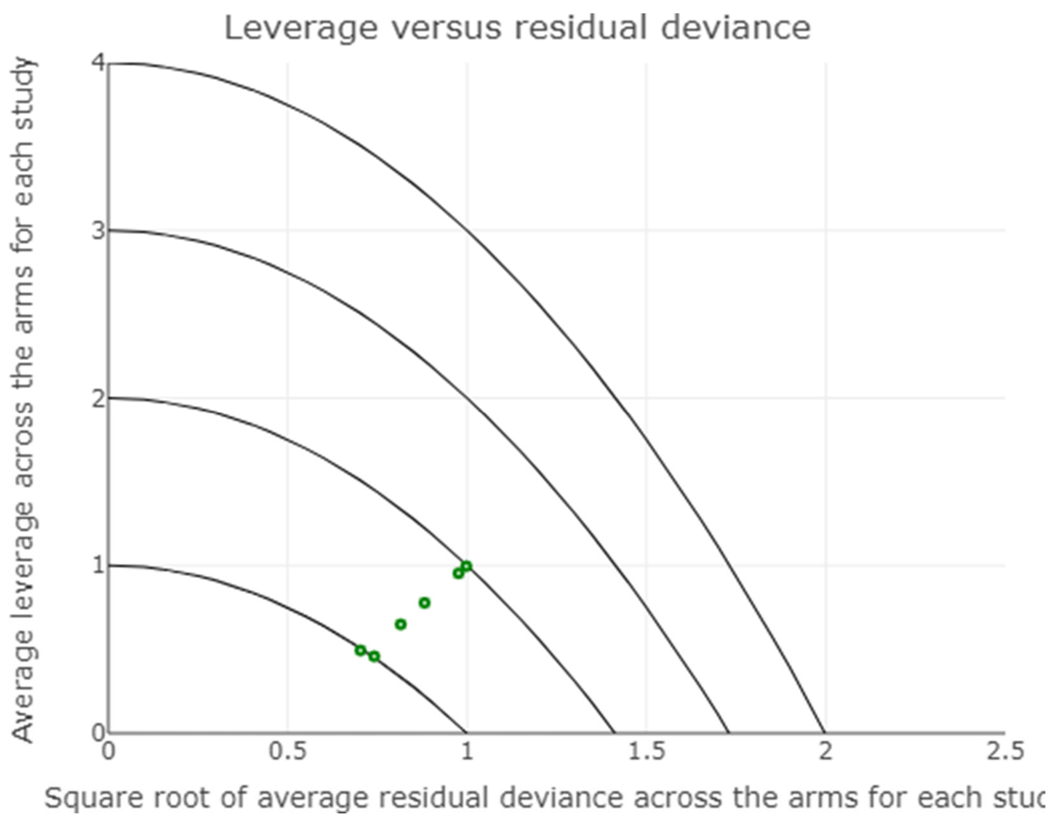

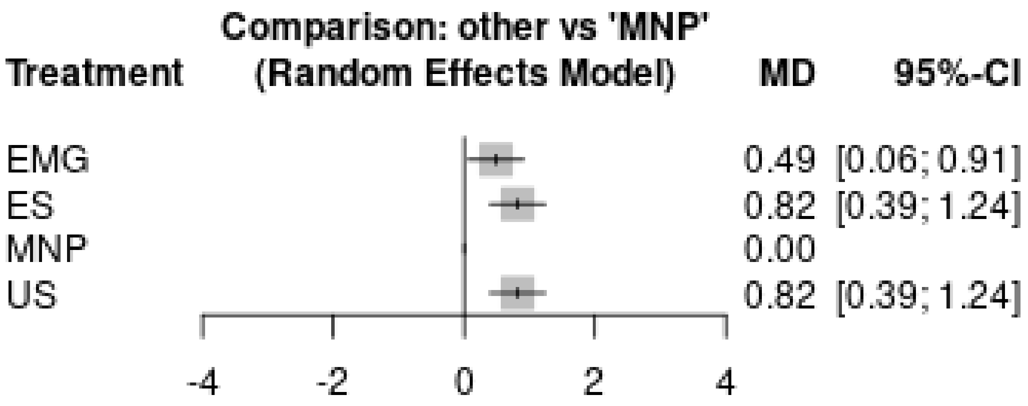

US	0.00 [0.00; 0.00]	0.47 [-7.24; 8.18]	0.88 [0.36; 1.41]
0.00 [0.00; 0.00]	ES	0.33 [0.32; 0.34]	0.80 [0.29; 1.32]
0.33 [0.32; 0.34]	0.33 [0.32; 0.34]	EMG	0.42 [-0.40; 1.24]
0.82 [0.39; 1.24]	0.82 [0.39; 1.24]	0.49 [0.06; 0.91]	MNP
